# Alternative Processing of the U2 Small Nuclear RNA Produces a 19–22nt Fragment with Relevance for the Detection of Non-Small Cell Lung Cancer in Human Serum

**DOI:** 10.1371/journal.pone.0060134

**Published:** 2013-03-20

**Authors:** Julien Mazières, Caroline Catherinne, Olivier Delfour, Sandrine Gouin, Isabelle Rouquette, Marie-Bernadette Delisle, Grégoire Prévot, Roger Escamilla, Alain Didier, David H. Persing, Mike Bates, Bernard Michot

**Affiliations:** 1 Service de Pneumologie, Hôpital Larrey, CHU de Toulouse, Université de Toulouse III (Paul Sabatier), Toulouse, France; 2 Cepheid Europe, Maurens-Scopont, France; 3 Service d'anatomie pathologique, Hôpital Rangueil, CHU de Toulouse, Toulouse, France; 4 Cepheid USA, Sunnyvale, California, United States of America; Sun Yat-sen University, China

## Abstract

RNU2 exists in two functional forms (RNU2-1 and RNU2-2) distinguishable by the presence of a unique 4-bases motif. Detailed investigation of datasets obtained from deep sequencing of five human lung primary tumors revealed that both forms express at a high rate a 19–22nt fragment (miR-U2-1 and -2) from its 3′ region and contains the 4-bases motif. Deep sequencing of independent pools of serum samples from healthy donors and lung cancer patients revealed that miR-U2-1 and -2 are pervasively processed in lung tissue by means of endonucleolytic cleavages and stably exported to the blood. Then, microarrays hybridization experiments of matched normal/tumor samples revealed a significant over-expression of miR-U2-1 in 14 of 18 lung primary tumors. Subsequently, qRT-PCR of miR-U2-1 using serum from 62 lung cancer patients and 96 various controls demonstrated that its expression levels identify lung cancer patients with 79% sensitivity and 80% specificity. miR-U2-1 expression correlated with the presence or absence of lung cancer in patients with chronic obstructive pulmonary disease (COPD), other diseases of the lung – not cancer, and in healthy controls. These data suggest that RNU2-1 is a new bi-functional ncRNA that produces a 19–22nt fragment which may be useful in detecting lung cancer non-invasively in high risk patients.

## Introduction

The exploration of the non-protein-coding RNA (ncRNA) transcriptome in human cells and tissues has long been focused on profiling microRNAs. The emergence of high-throughput Next Generation Sequencing technologies (NGS) has allowed the identification of new types of ncRNAs and paved the way for the study of their functional associations [1]. Most novel ncRNA species are now discovered using NGS approaches [2]–[5]. Since functional ncRNAs are thought to be protected from degradation by virtue of their association with proteins and packaging into particles, and therefore are stable, NGS offers the unique advantage of being able to detect the simultaneous expression of thousands of functional small RNA transcripts in a single tissue type, revealing a new level of complexity in the production of small ncRNAs in human cells, tissues, and organs under various physiological conditions [2], [4], [8]. The very high sensitivity of NGS methods has also allowed the discovery of previously undetected ‘isomirs’ produced by post-transcriptional editing mechanisms, single-nucleotide non-template 3′ adenosine or uracil additions serving as one example [6], [7]. Moreover, it was recently shown that a single ncRNA can generate different products not simply through random degradation but via specific tuning of the multiple steps of processing involving Dicer [9] or other enzymes [10], [11]. Such alternative ncRNA processing could explain how a single primary transcript ncRNA might subserve more than one function, analogous to the alternative splicing of pre-mRNA transcripts. Thus, the growing collection of bi-functional ncRNAs is offering a new view of human biology as seen through the lens of NGS.

These newly discovered classes of small RNA produced by ncRNAs with another function share some features in common with microRNA. There is now extensive evidence that several tRNAs produce tRNA-derived regulatory small RNAs (smRNAs) [9], [11]–[13]. Abundant small RNAs 18–22 nt in length, are processed from both the 5′ and 3′ ends of mature tRNAs, as well as from the 3′ trailer regions of pre-tRNAs. The mature 3′ population (also termed Type I) was found to be Dicer dependent and complexed with Argonautes 1–4. In contrast, the pre-tRNA 3′ trailer population (Type II) is Dicer independent and seemingly involves the action of RNase Z. In addition, the type II RNAs were found to be complexed with Argonautes 3 and 4 and to a much lesser extent to Argonautes 1 and 2 [13]. Concentrated in nucleoli, several snoRNAs function in the processing of ribosomal RNAs (rRNA) during ribosome subunit biosynthesis and assembly. However most of them serves as guide for enzymatic modification of target rRNAs and spliceosomal U6 snRNAs at nucleotides selected by RNA:RNA antisense interactions [14], [15]. SnoRNAs are classified in two groups, the C/D box snoRNAs which catalyze 2′ O ribose methylation [16], and the H/ACA box snoRNAs which introduce pseudouridine modifications [17]. Recent bioinformatics analyse of small RNA libraries have suggested the existence of smRNAs derived from certain of the snoRNAs following processing [18]–[21]. H/ACA and C/D box derived ncRNAs use divergent biogenesis pathways depending on the type of precursor snoRNA being processed [21]–[23]. H/ACA-derived ncRNAs are predominantly 20–24 nt in length and originate from the 3′ end. Their production is independent of Drosha, but requires the action of Dicer [18]. In contrast, the C/D derived ncRNAs are either 17–19 nt or >27 nt in length and predominantly originate from the 5′ end [21]. The great variety of their secondary structures, suggests Dicer independent processing based on Ago2 endonuclease activity similar to pre-miR-451 processing [24]. Several examples of smRNAs derived from tRNA or snoRNA precursors have already been shown to have possible biological functions [9], [11], [27], [28].

Short reads are also produced from a wide variety of other types of structured ncRNAs like 7SL, 5S, Y1 and Y3 [25]. The vault RNAs which are involved in multidrug resistance and intracellular transport in humans have recently been demonstrated to produce a group of ∼23-nt RNAs in a Drosha-independent but Dicer-mediated processing step [26]. One of the vault-derived ncRNAs is bound to Argonaute proteins and mediates mRNA cleavage, mimicing it key micro-RNA-like features.

All of these examples suggest that alternative processing of ncRNAs may represent an important mechanism by which functional diversity for ncRNAs is achieved, and furthermore implies that the new layer of regulatory influence and control imposed by ncRNAs on gene expression may be fundamental to biology. One can only imagine the potential impact of smRNAs derived from ncRNAs with another function on the expression of metabolic intermediates in different physiologic states, as well as the influence on key mediators of signal transduction in altered pathophysiologic states, such as cancer.

In contrast to snoRNAs and tRNAs, little is known about snRNAs which are components of the spliceosome complex, directing the accurate removal of intronic sequences from pre-mRNAs [29]. However, recent NGS experiments suggested that spliceosomal snRNAs U1, U2, U6 and U12 accumulate small RNA sequences [25], while immunoprecipitation with Argonaute proteins allowed the identification of Ago-bound RNA fragments from U1, U2 and U12 [30]. More recently, a fragment of RNU2-1 was shown over-expressed in pancreatic and colorectal cancers relative to normal tissue from those organs [31]. To better understand whether the expression of small ncRNA fragments resulting from alternative processing are associated with the presence of disease, we measured the levels of the fragments produced by the snRNA U2 by several methodologies in the lung tissue and serum of patients with lung cancer, and compared them to the RNU2 levels in the serum of patients at risk for lung cancer (patients with COPD) as well as normal healthy control subjects. RNU2 is one of the most highly expressed ncRNAs and it is preferentially expressed in lung tissue [32]. Combining NGS, microarray hybridization experiments and qRT-PCR, we performed a detailed analysis of RNU2-derived smRNAs in lung primary tumors, paired adjacent normal lung tissue from the same patients, and serum specimens from lung cancer patients and healthy control subjects, as well as controls with other lung diseases – not cancer. The results suggest that miR-U2, a 19–22nt product of RNU2 alternative processing, allows the discrimination of patients with lung cancer from those with COPD but no cancer, and raises the possibility that serum-based measurements of miR-U2 levels might be used as a non-invasive, cost-effective, early screening tool to select patients for further evaluation with advanced imaging diagnostics like spiral CT scanning.

## Materials and Methods

### Samples Collection

Tissue specimens and serum samples were collected at Hospital Rangueil and Hospital Larrey, Toulouse, France. Written informed consent was obtained from all individuals before their enrollment in this study, and the study was approved by appropriate institutional review boards. All of the records were anonymized to protect individual confidentiality. Clinical data were collected for each individual at the time of tissue or blood collection. Clinical stage was determined according to the 7^th^ International Association for the Study of Lung Cancer staging system [33]. For eight patients out of eighteen who were operated on for lung cancer, paired primary tumor and distal normal tissue specimens were collected. The blood of lung cancer patients was collected at the time of diagnosis but prior to tumor resection or any specific treatment. Five ml of blood were collected from each individual in SST tubes and immediately processed. The tubes were mixed by a few inversions, and then put at room temperature for 30 min for clotting. Tubes were then centrifuged (1000 g at 4°C) for 10 min. Finally, the serum fraction was aliquoted in 1 ml tubes and immediately stored at −80°C until the time of use. 45 healthy control samples were also purchased from Asterand and processed in an identical manner. We organized this cohort into five groups. Three control groups contain individuals without a lung cancer: 1) only people without any symptoms of disease at the time of blood draw. They can be considered “healthy” individuals. 2) patients with a lung disease which is not COPD. This group is comprised mainly of persons suffering from asthma, bronchitis, and pulmonary infections. 3) COPD patients without any evidence of lung cancer at the time of the blood draw. Patients with a lung cancer were split in two groups: 1) all patients with lung cancer without any COPD symptoms, and 2) patients suffering from both a lung cancer and COPD.

### Extraction-Purification of RNA

RNA was extracted from 0.5 ml of serum using the Qiagen miRNeasy mini kit, according to the manufacturer's instructions. A spike-in control (Cel-miR-39) was added after the first denaturing step.

Archived or freshly snap-frozen tissue specimens were homogenized by mortar and pestle in TRIzol® Reagent Life Technologies (Carlsbad, CA) and RNA was further extracted according to the manufacturer's protocol. The small RNA fraction was extracted using the Flash PAGE Fractionator (Ambion). All microRNA samples were diluted in RNase-free water and stored at −80°C.

### Deep sequencing and data treatment

Library preparation and sequencing experiments using RNA purified from tissue or serum specimens were performed by Fasteris (Switzerland). Briefly, RNA fragments (16–30 nt or 25–50 nt) were gel purified followed by ligation of single-stranded RNA 3′ and 5′ adapters. An acrylamide gel purification step was performed prior to reverse transcription and PCR amplification to generate the DNA colony template library. cDNAs were gel purified and the library was quantified before dilution to10 nM. The diluted cDNA libraries were then sequenced using the Illumina HiSeq technology. The sequence reads were processed by a combination of bioinformatics algorithms and manual curation to remove the 5′ and 3′ end adapters (Table S7). Only the reads of 19nt long or longer following adapter removal were retained for analysis. The 5′ and 3′ ends mismatches were trimmed until perfect-match reads. Reads from the different libraries were normalized using the total number of reads in each library and expressed as reads per million (RPM) for the purpose of comparing relative expression levels. Only reads with a unique match to the human genome were used (NCBI build 37). Thus, reads mapping to functional RNU2 genes map to no other genomic location. No internal mismatches were allowed. For higher confidence, only RNA with a minimum of five RPM were considered for further analyse.

### Microarrays Hybridization Experiments

Nexterion (Schott) microarray glass slides were used as the solid support for the microarray. Custom in house designed oligonucleotides were spotted into their surface. The labeling of the microRNA was adapted from Castoldi et al. 2006 [34]. The labeled microRNA fraction was hybridized to the spotted arrays using the Discovery hybridization station (Ventana, Tucson, AZ). The arrays were scanned using the Axon scanner (Molecular Devices, Sunnyvale, CA) and data were collected using Genepix software. Six in house designed spike-in internal controls were used to normalize the data. These synthetic RNA controls were added to the total RNA fraction before hybridization. All sequences for which the intensity of the spot was higher than the mean local background intensity plus 1.5 times its standard deviation were categorized as expressed microRNAs. Statistical normalization of the data was done by computing the Log_2_ ratio where the Log_2_ ratio  =  average intensity signal of the duplicated spots/median intensity of all internal controls for the block. The normalization was done per block to avoid non-homogenous labeling (Table S4). Microarray data were analyzed using the R bioconductor package [35]. The relative expression of each microRNA was calculated using the 2^−ΔΔCT^ method [36]. The “MeV 4.8.1” (MultiExperimentViewer) [37] was used for hierarchical clustering analysis, where selected miRNAs were clustered using Euclidean distance.

### qRT-PCR of miR-U2 expression

Expression levels of miR-U2-1 were assessed by qRT-PCR using the Exiqon custom LNA^TM^ primers (Exiqon, Vedbaek, Denmark) according to the manufacturer's instructions. Although the primers were design to detect the isoform UGGAUUUUUGGAGCAGGGAG the Exiqon primers allow the detection of the other isoforms (Tables S5 and S6). Experiments were performed in triplicate. Because of the lack of serum housekeeping small ncRNAs, we normalized microRNA concentration to the initial volume of serum (500 µl). An exogenous spike-in control (Cel-miR-39) was also used in order to check the robustness of the results. We calculated the ΔCt value matrix for each sample by subtracting the threshold cycle number (Ct) value for miR-U2 from the Ct value of Cel-miR-39. A one-way analysis of variance (ANOVA) was used for statistical analysis comparing the sample populations and the control groups. The area under the curve (AUC) was calculated for receiver operator curves (ROC). Results determined using normalization to the Cel-miR-39 spike-in and normalization to volume of serum were compared.

## Results and Discussion

### Lung primary tumors highly express a 19–22nt ncRNA processed from RNU2

**Figure 1:**
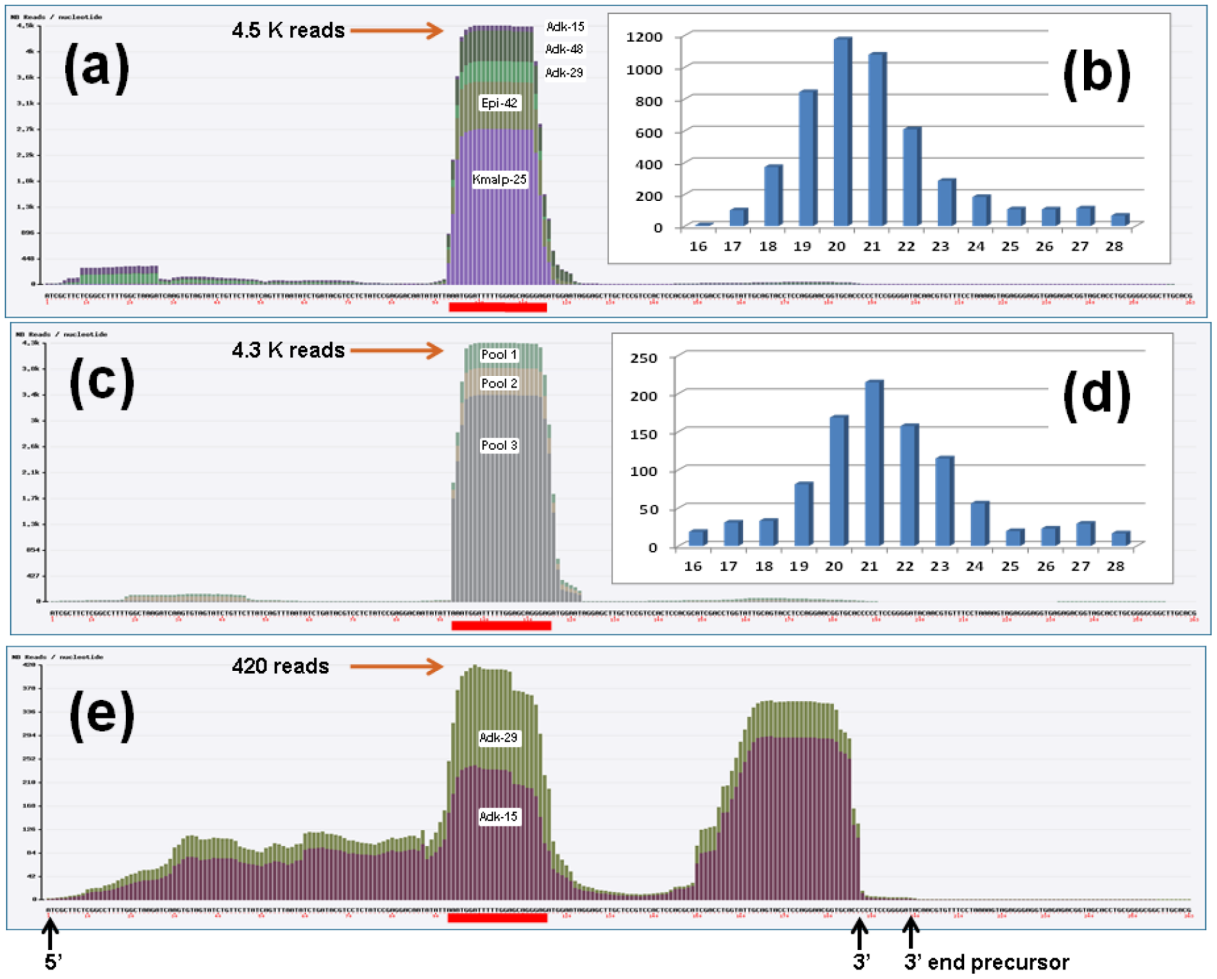
Normalized number of reads mapped along the RNU2-1 gene. Small reads detected in Primary tumors (a) and serum specimens (c). Long-reads detected in Primary tumors (e). The color code shows the number of normalized reads detected in each library. The red line locates miR-U2. The number of reads according to their size is given in (b) for the primary tumors, in (d) for the serum specimens.

The human genome encodes two functional U2 snRNA genes, RNU2-1 and RNU2-2 that are located at 17q21.31 and 11q12.3 respectively. The genomic organization of the two RNU2 genes differs radically. RNU2-2 is present at a single copy whereas RNU2-1 is organized in a cluster of 6 to ∼30 tandemly repeated copies spanning 30 to 150 kb. Each repeat unit is 6.1 kb long, contains a single RNU2-1 gene and is highly conserved with other repeat units. Although RNU2-1 differs in gene copy number from individual to individual, the arrays are stably inherited, subject to dosage compensation and transcribed at an unusually high rate. In order to discover if small ncRNAs might be effectively processed from the two functional RNU2 genes and to delineate such products precisely, we sequenced the small RNA transcriptome of five human lung cancer primary tumors as well as three pools of serum samples, one from healthy donors and two from lung cancer patients (Table 1). Sequencing related samples in parallel minimizes the rate of false discovery of ncRNAs. ncRNAs that are truly over-expressed would be expected to be found in multiple independent samples. In a first step, we mapped the reads sequenced from seven libraries prepared from primary tumors, five short sized (16–30nt) and two long sized (25–50nt) directly onto the RNA sequence of the functional RNU2 genes. The RNU2-1 locus on chromosome 17 was actually removed from the human genome assembly since version Build 37 (NCBI Annotation Information Gene ID: 6066, updated on 20-Apr-2012). The large majority of reads from the five short sized libraries maps at a unique location between positions 93 and 114 of RNU2-1 (Fig. 1a–b). The most highly expressed reads ranges from 19 to 22nt in length. This RNA fragment is called miR-U2-1. Remarkably, small RNA sequencing of the three pools of serum specimens demonstrated that miR-U2-1 is present in the blood circulation in both lung cancer patients and healthy subjects, revealing its pervasive expression (Fig. 1c–d). These data suggest that miR-U2-1 is actively exported rather than passively released into the circulation. Only a subset of miRNAs has indeed been detected outside cells [38]–[41], thus miR-U2-1 may represent an additional member of this class.

**Table 1:** Deep sequencing of lung cancer primary tumors and serum specimens.

							Gel Purification Size:		Gel Purification Size:	
							16–30nt		25–50nt	
Patient ID	Histology	Gender	Age	Smoking history	TNM staging	Stage group	Total Numberof Reads	Reads mapped to the human genome	Total Number of Reads	Reads mapped to the human genome
**Primary Tumors**										
Adk-15	Adc	M	64	Current Use	T2 N0 M0	IB	88 683 943	74 395 594	74 301 140	68 096 627
Adk-29	Adc	M	79	Current Use	T4 N2 M1	IV	97 500 752	74 246 018	80 178 518	73 712 260
Adk-48	Adc	F	56	na	T1b N0 M0	IA	25 350 247	19 914 778	/	/
Epi-42	SCC	M	59	na	T3 N1 M0	IIIA	25 265 410	19 759 343	/	/
Kmalp-25	SCC	M	82	Never Used	T2 N0 M0	IB	87 397 444	74 467 579	/	/
**Serum Pool 1 – Lung Cancer**										
52825	SCC	M	56	Current Use	T1 N0 M0	IA	23 891 527	20 118 929	/	/
50532	Adc	M	53	Previous Use	T1 N0 M0	IA				
52840	Adc	M	56	Current Use	T2 N1 M0	IIB				
**Serum Pool 2 – Controls**										
31465	control	M	25	Never Used	/	/	26 141 074	23 975 546	/	/
31504	control	F	34	Never Used	/	/				
32747	control	M	31	Never Used	/	/				
**Serum Pool 3 – Lung Cancer**										
45415	SCC	F	75	Never Used	T1 N0 M0	IA	19 236 794	13 065 962	/	/
47041	SCC	M	62	Current Use	T1 NX M0	IA				
49133	SCC	M	72	Current Use	T2 N0 M0	IB				
49731	Adc	F	67	Never Used	T1 NX M0	IA				
50542	SCC	M	55	Current Use	T1 N0 M0	IA				
51088	Adc	M	47	Current Use	T2 NX M0	IB				
51952	Adc	F	61	Never Used	T1 N0 M0	IA				
52017	SCC	F	65	Never Used	T2 N1 M0	IIB				
55184	SCC	M	43	Current Use	T3 N0 M0	IIB				
55185	Adc	M	55	Previous Use	T3 N0 M0	IIB				
55189	Adc	F	59	Never Used	T2a N0 M0	IB				
55879	SCC	M	54	Previous Use	T1a N0 M0	IA				
56318	Adc	M	50	Never Used	T2a NX M0	IB				
56320	SCC	M	55	Never Used	T1b N1 M0	IIA				
56330	SCC	M	49	Never Used	T3 N0 M0	IIB				
45415	SCC	F	75	Never Used	T1 N0 M0	IA				
47041	SCC	M	62	Current Use	T1 NX M0	IA				
49133	SCC	M	72	Current Use	T2 N0 M0	IB				

The number of reads detected in each condition is indicated. Adc and SCC stand for adenocarcinoma and squamous cell carcinoma respectively. We selected only reads matching the three following criteria: 1) reads are 17nt long or more; 2) at least 17 consecutive nucleotides match perfectly with the human genome (GRCh37); 3) the nucleotide at the 5′ end of reads maps the human genome. Two types of libraries were built. The short one was built after a gel purification of RNA fragments between 16 to 30 nt long; the long one after a gel purification of RNA fragments between 25 to 50 nt long.

**Figure 2:**
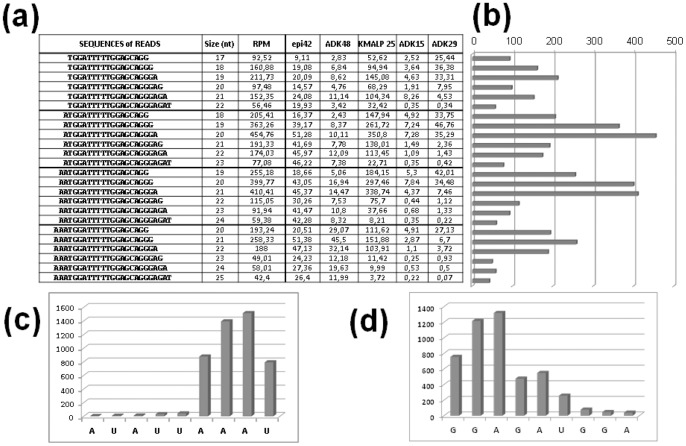
Most represented isoforms. (a) Indicates the sequence of each read detected and the normalized number of each read is given for each tumor specimen. (b) The total number of each type of read is shown as an histogram. Histograms (c) and (d) display the number of reads at the diverse 5′ starts and 3′ ends of miR-U2 respectively.

Among the population of reads present in primary tumors as well as in serum, we observed an accumulation of RNA fragments starting at position “A-94” or “A-95” and terminating at position “G-113” or “A-114” (Fig. 2). This limited number of 5′ and 3′ end reads corresponds to a definite and small population of variants or isomirs, a commonly feature of microRNAs. Together, these miR-U2-1 isomirs represent more than 75% of the reads matching exclusively to this region of RNU2-1. Their expression levels are in the range of canonical microRNAs like miR16, miR-17, miR-200a or miR-451 (Table S1). This significant accumulation of a unique and precisely cleaved RNA fragment from RNU2-1 represents clear evidence favoring the hypothesis that specific processing events produce a new functional ncRNA, miR-U2-1, rather than the alternative explanation, that these are the random products of RNA degradation. These results further indicate that miR-U2-1 is protected against endo- and exo-nucleolytic RNase digestion by virtue of packaging in complex nucleoprotein particles. miR-U2-1 may also be wrapped in small membranous particles (exosomes, microvesicles, apoptotic bodies) before export to the circulation. The majority of microRNAs detectable in serum are indeed concentrated in exosomes [42], although some may simply be associated with proteins [43]. Taken together, this suggests that the processing events producing miR-U2-1 represent essential biological processes in human.

The sequences of the RNU2-1 and RNU2-2 genes (187 nt) are highly conserved. The differences between them are restricted to two point mutations located at the 3′ end (positions 170 and 187) and to a 4-base mutation located at positions 108-111 (Fig. S1). Remarkably, RNU2-2 expresses also the same region with an identical size (Fig. S2). The higher number of reads detected for miR-U2-1 in comparison with miR-U2-2 is consistent with the RNU2-1's higher genomic copy number. Fortunately, the 4-base difference (GCAG and AAUA in RNU2-1 and RNU2-2 respectively) distinguishing the two U2 genes is located within the miR-U2 sequences, permitting discrimination of the two products. This polymorphism does not appear to have any perceptible impact on processing, suggesting similar biogenesis mechanisms for miR-U2-1 and miR-U2-2. However, the differences in sequence between miR-U2-1 and miR-U2-2 may reflect further functional diversification, perhaps facilitating a fine tuning of regulatory function through differential target selection or protein factors binding.

The use of NGS technology is not without its pitfalls. It is well established that small RNAs can be inadvertently cross-mapped to the wrong genomic region. RNU2 genes are a complex family comprising more than 50 pseudogenes which makes cross-mapping errors distinctly possible. It has recently been reported that two immunoprecipitated argonaute-associated small RNAs of 23nt and 30nt were mapped to two different U2 pseudogenes [30], to positions 168–187 of the U2 pseudogene encoded by chromosome 6 and to positions 95–125 of the U2 pseudogene encoded by chromosome 10 respectively. These two fragments map the functional U2-1 RNA with 100% identity. We had earlier noted that the RNU2-1 locus on chromosome 17 was actually removed from the Human Genome Sequence Assembly Build 37 (NCBI Annotation Information Gene ID: 6066, update 20-April-2012), and this is the most probable explanation as to why these RNA fragments mapped to these pseudogenes rather than the functional RNU2 gene itself. Similarly, two short microRNAs (19 nt), miR-1246 and miR-1290, match miR-U2-1 with 100% identity and one mismatch respectively [44]. Mapping reads from our five short libraries to the respective precursors of these two microRNAs [45] revealed unambiguously that the two putative precursors of miR-1246 and miR-1290 are the result of false-mapping. The two proposed mature microRNAs are actually truncated versions of miR-U2-1. This was indirectly confirmed for miR-1246, when attempts to sequence its precursor in the same tissue where miR-1246 was detected failed, suggesting that the supposed precursor does not exist [46]. This result was recently independently confirmed [31]. Regarding miR-1290 there are two possible genomic locations that could encode it (Fig. S3), however, in our experiments it is detected at the same level as the background noise associated with sequencing errors (Table S2). We conclude from these data that all results related to miR-1246 and miR-1290 can be assigned to miR-U2.

### A dual function for the RNU2 genes

**Figure 3:**
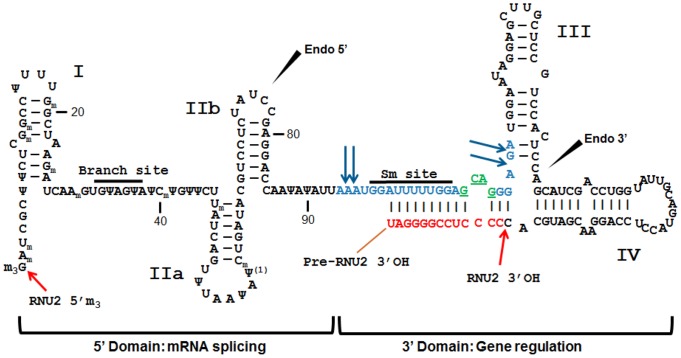
miR-U2 processing. The secondary structure folding of the RNU2 precursor is shown. Ψ locations are from [49]. Methylated nucleotides are indicated by the symbol “_m_”. Nucleotides in blue and green correspond to miR-U2-1 sequence. The green letters localize the four nucleotide mutation between miR-U2-1 and miR-U2-2. Black and red letters show RNU2 and precursor-specific RNU2 sequences respectively. Blue arrows point on the 5′ and 3′ ends of miR-U2 isomirs. The red arrow indicates the 3′end of RNU2. The two black arrowheads point on the two internal cleavage sites identified in this work.

The experimental results discussed thus far suggest a dual function for the RNU2-1 and RNU2-2 snRNAs. Their 5′ domain contains the mRNA branch site involved in mRNA splicing, while their 3′ domain encodes miR-U2 which contains the Sm binding site and could be involved in the regulation of gene expression. This functional partition of RNU2 correlates with the observed differences in structural features that exist in the molecule (Fig. 3). The 5′ portion contains an impressive number of post-transcriptional modifications including 14 pseudouridylations and 10 methylated nucleotides [47]–[49]. In contrast, no modifications are identified downstream of position 90 of RNU2, and this domain contains more extensive secondary structure, particularly when the precursor sequence is included. These striking structural dissimilarities are likely related to the respective differential functions of the two halves of RNU2. Although the 5′ domain is sufficient to induce mRNA splicing in vitro [50]–[52], there is no evidence either from the literature or from our NGS experiments that any part of the 5′ region of RNU2 accumulate in cells. This suggests an alternative processing mechanism that produces the full-size RNU2 on the one hand, and miR-U2 on the other.

In order to better understand the mechanism of miR-U2 splicing, we sequenced two cDNA libraries for ncRNAs longer than miR-U2 (25–50nt long) in order to identify potential intermediates (Table 1). The number of long-reads mapping to RNU2 is considerably lower than that seen in the small-size cDNA libraries, suggesting that the intermediate of maturation is rapidly degraded or trimmed to produce the final mature microRNA. Examining the distribution of reads (measured in RPM) along the pre-RNU2 sequence, we observe two peaks corresponding to two smaller fragments which accumulate (Fig. 1e). As expected, one peak maps to the mature miR-U2, while surprisingly the second maps to the 3′ end of the mature RNU2, a region which does not produce a stable ncRNA since we did not observe it in our sequencing results from the short-sized libraries. This RNU2 fragment corresponds exactly to the 30nt-long fragment binding Ago1 [30]. This suggests that the degradation of the 3′ end of RNU2 is impeded by the transient binding of Ago, but because the protection afforded by Ago 1 is temporary, it does not give rise to a stable ncRNA product. Alternatively, this product could be degraded in lung tissues because of the absence of additional factors, but could be expressed in other cell types or tissues. The decreasing number of reads of both sides of the two peaks reflects a degradation of the intermediates of maturation by trimming mechanisms from both ends, while the valley between them locates the position of an endonucleolytic cleavage site (endo 3′) in the vicinity of position 145 within stem IV (Fig. 1e and Fig. 3). In contrast, the 5′ half does not contain any fragment protected from degradation. Nevertheless, a second endonucleolitic cleavage site (endo 5′) appears to be located between positions 70 and 80 within stem IIb.

Considering the different potential processing pathways for RNU2, the most likely first step involves the 3′end cleavage at position 187. We can find no read matching the expected specific precursor, indicating an early and fast event. This first step involves a base-paring between the precursor specific sequence (positions 190–200) and positions 100–110 of RNU2 which is a key structural feature required for guiding the 3′ end processing [53]. Remarkably, this base-pairing occurs with the miR-U2 region and delineates precisely the two structurally and functionally distinct halves of RNU2 (Fig. 3). Then, in absence of Ago1-binding, the binding of Sm protein, likely in combination with other factors like U2A′ and U2B″, might stabilize the full length RNU2. In contrast, the Ago1 binding to miR-U2 sequences could induce a shift to the endonucleolytic cleavages pathway which cleaves at positions 70–80 and 145 respectively. Multiple and adjacent small RNA-binding sites for Ago1 are known to facilitate cooperative interactions that stabilize Argonaute binding [54]. Since Ago1 has no enzymatic activity, an RNase must be recruited for miR-U2 maturation, similarly to miR-like ncRNAs produced from snoRNAs and tRNAs. Finally, this transient precursor is rapidly trimmed from both ends to yield the mature miR-U2.

The exclusive binding of miR-U2 to Ago1 [30], as opposed to most of canonical microRNAs which also bind Ago2 [55], suggests a different role for miR-U2. Although the preponderance of the data indicates that microRNAs regulate gene expression in the cytoplasm and P-bodies, the four Argonaute proteins and a number of microRNAs have been recently discovered in the nucleus of human cells. The transport of small RNAs in complex with Ago proteins into the nucleus depends on Importin-8 [56]. This mechanism suggests their possible interaction with the chromatin and opens the exciting alternative of their direct involvement in affecting nuclear processes like splicing or transcription by targeting gene promoter regions. Interestingly, several recent studies revealed functional segregation between Ago1 and Ago2 [57] as well as a differential distribution in the nucleus in response to promoter-targeted siRNA during transcriptional gene silencing [58] suggesting that Ago proteins mediate the nuclear localization of the small RNAs. In addition to defining the biological activity of small RNAs, Ago proteins may also define the length of mature microRNAs [59].

This allows us to consider the possibility that miR-U2 might play epigenetic roles at the DNA level where it could act as a regulator of the expression of large chromosomal regions. It is indeed known that many promoters of tumor-suppressor genes show higher levels of methylation, suggesting a possible connection with cancer risk. miR-U2 could for example participate in guiding cytosine methylation, a process that is known to be responsive to environmental stimuli. The 4-nt difference between RNU2-1 and RNU2-2 could account for differences in target selection or the recruitment of specific protein factors, providing a mechanism by which regulatory influences could be fine tuned. Alternatively, miR-U2 could behave like a canonical microRNA. Recently, expression profiling of human miRNAs in colorectal tumors combined with miRNA-network analysis, identified an association between miR-1246 over-expression and the development of colorectal cancer [60]. Independent work revealed that miR-1246 is a target of p53 family members. TP53 induces the expression of miR-1246 which, in turn, reduces the level of DYRK1A resulting in a decrease in the induction of apoptosis [61]. As previously discussed, miR-1246 is very likely a false mapping of miR-U2, thus these results more probably concern miR-U2.

The balance between the two processing pathways producing on the one hand full length RNU2, and on the other hand miR-U2, is certainly under a precise regulatory control. This makes the level of miR-U2 dependent on Ago1 levels and/or binding efficiency, as well as on the other factors required for its accurate processing. The recent discovery that U2 is essentially expressed in lung tissue [32] and that Ago1 is involved in lung cancer [62], suggest the possibility that a deregulation of this balance could occur during the development of lung cancers.

### Circulating miR-U2 in serum is a potential new biomarker for lung cancer

**Figure 4:**
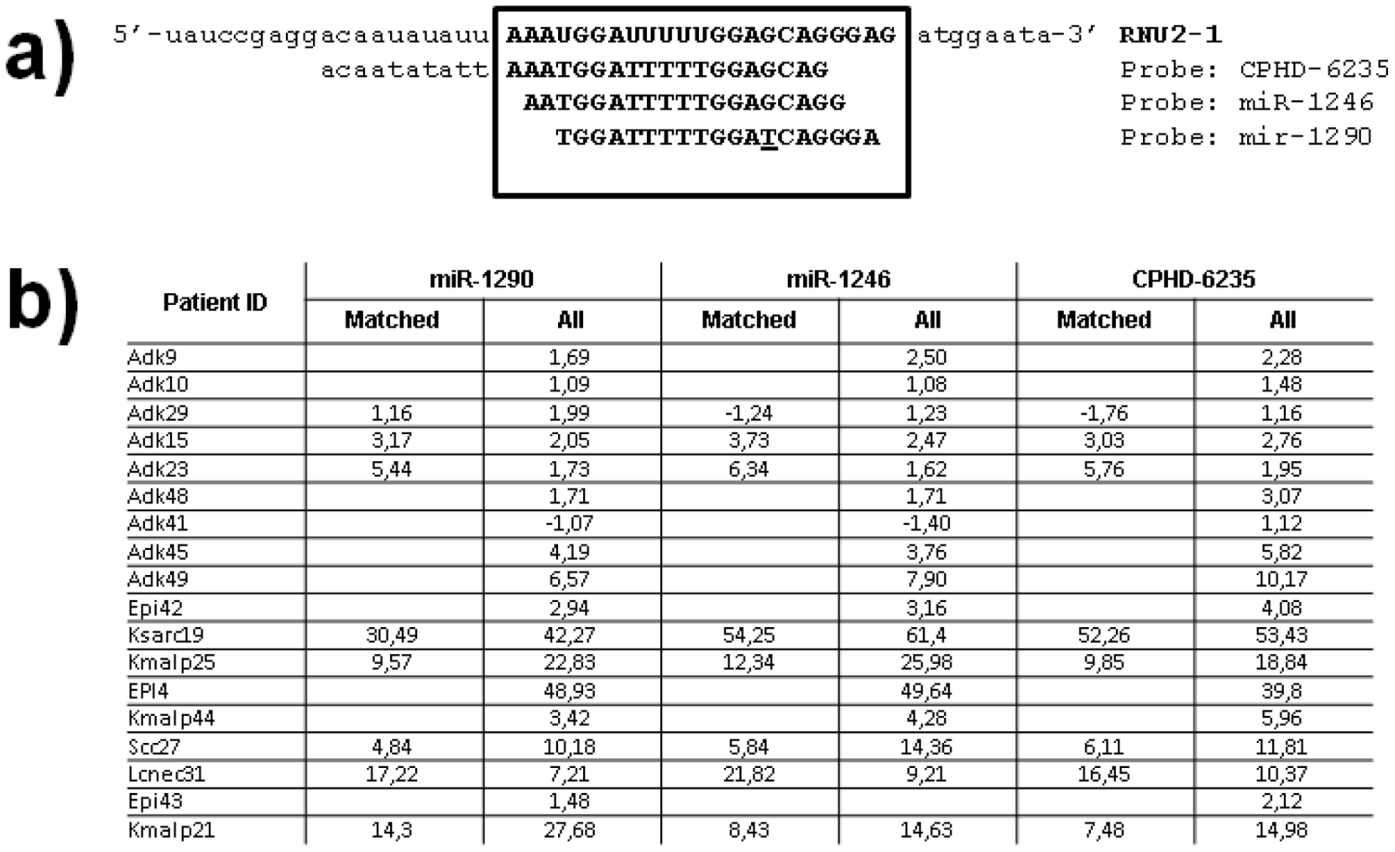
Microarrays profiling of miR-U2-1. In a) are indicated the three probes used for detecting miR-U2-1. The boxed region corresponds to the miR-U2-1 longest and most expressed isoforms; upper case letters indicate nucleotides belonging to miR-U2-1, while lower case letters are for nucleotides belonging to the RNU2-1 gene. The red nucleotide points on the difference between miR-1290 and miR-U2-1; b) gives the fold changes calculated between primary tumors and matched normal tissue (column “matched”) or between primary tumors and the averaged value of all normal tissues (column “All”).

In our attempts to identify microRNAs that are significantly over- or under-expressed during lung cancer initiation and development we had profiled 19 primary tumors as well as eight adjacent paired normal lung tissues (Table 2) on custom microarrays with 837 known microRNAs (miRbase 11) and a proprietary collection of more than 2,300 microRNA candidates (unpublished results). Among the probes spotted, three targeted miR-U2-1 (Fig. 4a). Probes-1246 and -1290 correspond to miR-1246 and miR-1290 sequences respectively, while CPHD-6235 is longer covering 10 nt upstream of miR-U2-1. Because miR-1246 and miR-1290 cross-map to miR-U2-1, the signal detected by their respective probes corresponds exclusively to hybridization with miR-U2-1. The probe CPHD-6235 also hybridizes exclusively to miR-U2-1. The analysis of the 8 matched samples reveals a very similar expression profile for the three probes, and a significant over-expression (>3 fold) of miR-U2 is observed in the primary tumors relative to the adjacent normal tissue for seven of eight patients (Fig. 4b). Interestingly, among the eight normal lung tissues, the expression profile of most of the microRNA candidates analyzed remained remarkably consistent (Fig. S4). This was particularly true for the three probes matching miR-U2. This allowed us to use the average expression value issued from these three probes in normal tissues in comparative analyses of miR-U2 expression in the primary tumors for which no matched normal tissue was available. (Fig. 4b). Using this method, the fold changes for the 11 primary tumors for which matched adjacent normal tissue was not available showed an over-expression (>x2) in 12 individuals out of 19 (i.e. 63%) with probes corresponding to miR-1290 and miR-1246 sequences, and 14 individuals (i.e. 74%) with the probe CPHD-6235. These results suggest an association between over-expression of miR-U2 and lung cancer when compared with normal lung tissue.

**Table 2:** Demographic and histopathologic data for lung cancer patients enrolled in the microarray-based discovery cohort.

ID	Type of tissue profiled	Age at Excision	Gender	Smoking Status	Clinical Diagnosis	AJCC/UICC Stage	AJCC/UICC Stage Group
adk41	Primary Tumor	62	Female	Current Use	Adenocarcinoma	T2N0M0	IB
adk10	Primary Tumor	49	Male	Current Use	Adenocarcinoma	T3N0	
adk45	Primary Tumor	61	Female	Current Use	Adenocarcinoma	T3N2M0	IIIB
adk15 (1) (2)	Primary Tumor + ANT	61	Male	Current Use	Adenocarcinoma	T2N0M0	IB
adk49	Primary Tumor	66	Male	Never Used	Adenocarcinoma	T3N2	
adk23 (1)	Primary Tumor + ANT	37	Female	na	Adenocarcinoma	T3N0M0	IIB
adk29 (1) (2)	Primary Tumor + ANT	76	Male	Current Use	Adenocarcinoma	T4N2M1	IV
adk48 (1)	Primary Tumor	56	Female	na	Adenocarcinoma	T1bN0M0	IA
adk9	Primary Tumor	55	Male	Current Use	Adenocarcinoma	T1N0	
epi4	Primary Tumor	78	Male	Current Use	Squamous cell carcinoma	T2NX	
epi42 (1)	Primary Tumor	59	Male	na	Squamous cell carcinoma	T3N1M0	IIIA
epi43	Primary Tumor	72	Male	Current Use	Squamous cell carcinoma	T1N1	
kmalp21	Primary Tumor + ANT	66	Female	na	Squamous cell carcinoma	T3N1	
kmalp25 (1)	Primary Tumor + ANT	80	Male	na	Squamous cell carcinoma	T2N0M0	IB
kmalp44	Primary Tumor	70	Male	Never Used	Squamous cell carcinoma	T2N0	
ksarc17	ANT	53	Male	na	Carcinome Sarcomatoide	T2N0	
ksarc19	Primary Tumor + ANT	59	Male	na	Carcinome Sarcomatoide	T4N1	
lcnec31	Primary Tumor + ANT	62	Male	na	Large cells neuroendocrine car.	T3N2	
scc27	Primary Tumor + ANT	na	Female	na	Small cells carcinoma	T2N0	

ANT stands for Adjacent Normal Tissue. The number between parentheses indicates primary tumors which were also sequenced: (1) short size fragments (16–30nt long); (2) long size fragments (25–50nt long).

**Figure 5:**
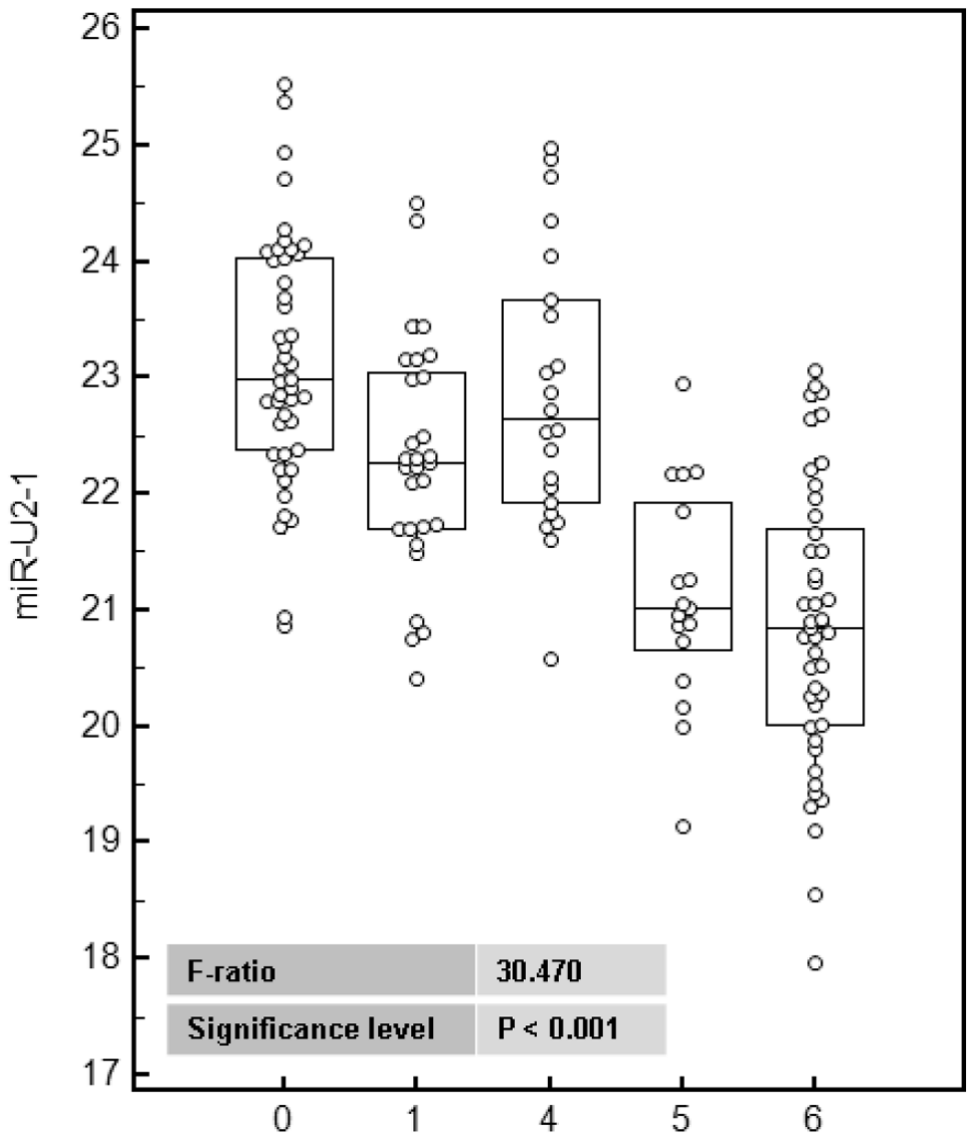
Anova of miR-U2 in the serum of the cohort. “0” stands for the “healthy” group of control individuals; “1” for the “lung diseases not-cancer not-COPD” group; “4” for the “COPD not-lung cancer” group; “5” for COPD patients with a lung cancer; “6” for lung cancer patients without COPD.

**Figure 6:**
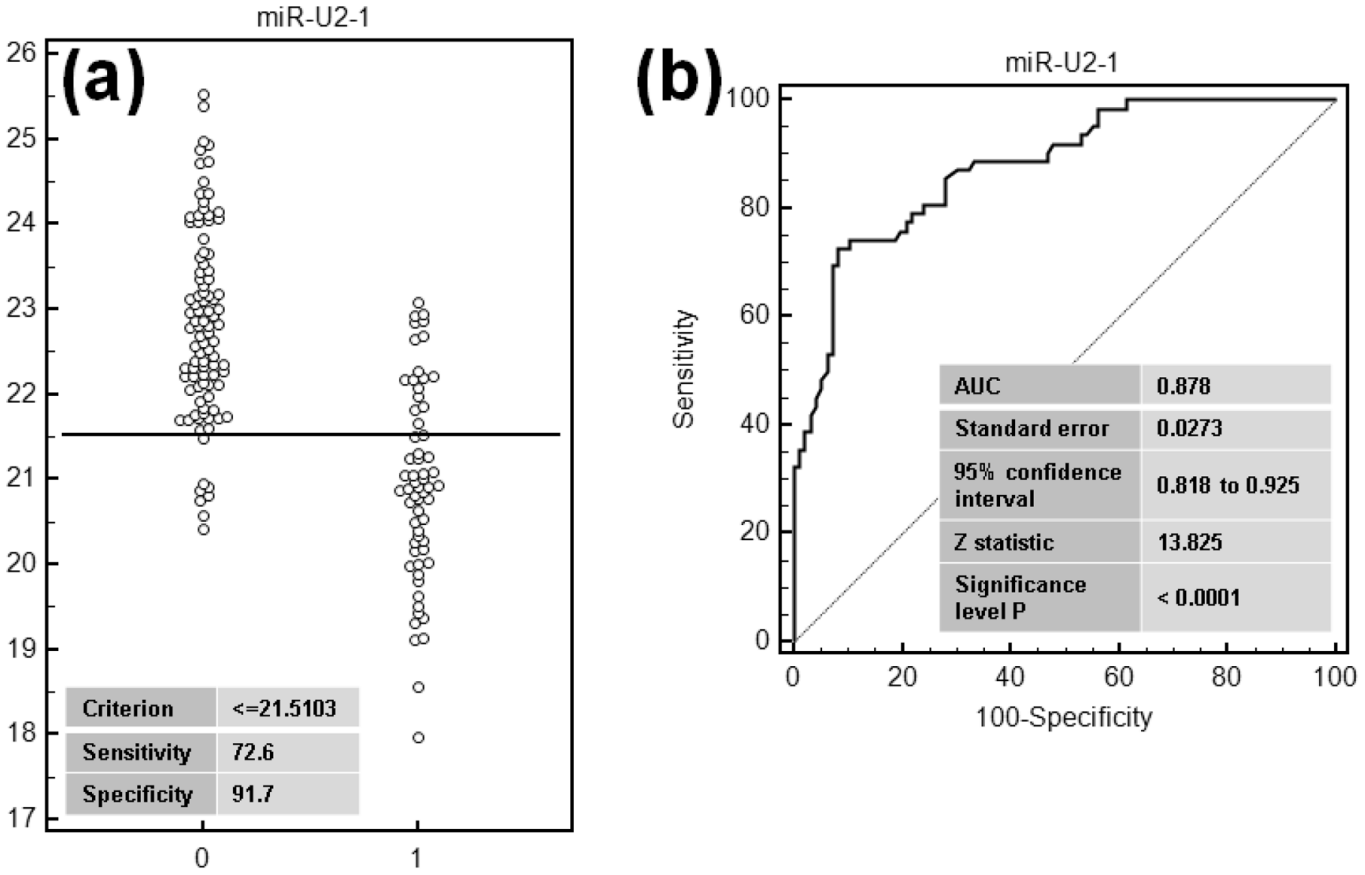
Roc (Receiving Operator Curves) of miR-U2 in the serum of the cohort. “0” stands for control individuals, “1” for lung cancer patients.

Numerous studies have shown that the level of extracellular and circulating miRNAs correlate with disease. RNU2 is expressed at much higher level in lung than in most of other tissues [32] and miR-U2 is efficiently exported into the circulation (our results and [46]) suggesting that changes in miR-U2 levels resulting from disease of the lung may indeed be detectable in serum. This prompted us to quantify miR-U2-1 in the serum of lung cancer patients. As miRNA have been shown to be deregulated in many lung diseases, including infection, inflammation or neoplasms, we included in our cohort, individuals with a variety of lung diseases to ensure the specificity of miR-U2-1 for lung cancer. We organized this cohort in five groups (Table 3, for detailed information see the material and methods section and Table S3). In this exploratory phase, we first compared the expression levels of miR-U2-1 among the five groups (Fig. 5). The ANOVA test based on Ct values using normalization to the volume of serum showed that miR-U2-1 is significantly over-expressed between patients with lung cancer and control subjects without lung cancer (P<0.001). These results suggest that lung diseases excluding cancer have no significant effect on the biogenesis of miR-U2-1 or its export to the blood. In order to check the robustness of these results we ran the ANOVA test on the ΔCt values obtained after normalization to the Cel-miR-39 spike-in control (Fig. S5). The results show a very similar stratification between the three non-cancer groups and the two lung cancer groups (P value <0.001). In comparison to the 96 controls, we are able to identify the 62 lung cancer patients with a sensitivity and specificity of 72.6% and 91.7% when data are normalized to the volume of serum (Fig. 6a), and 79% and 79.2% respectively after a normalization of Ct values to the spike-in control (Fig. S6a). The area under the curve (AUC) of the receiver operator characteristic (ROC) plot (Fig. 6b and Fig. S6b) is 8.78 and 8.38 when normalization is performed to the serum volume or to the spike-in respectively. Because the detection of this disease in its very early stage of development is a critical challenge for developing a reliable diagnostic tool, we included in our cohort six stage-I patients and seven stage-II patients. Although this number of individuals is not sufficient for drawing a definitive conclusion, we observed a similar over-expression at stages I, III and IV of lung cancer patients but a significant decrease of the amount of miR-U2-1 at stage II (Fig. S7). This suggests that the transition from stage I to stage II induces or depends of very specific molecular mechanisms and genes regulation. This significant increase of miR-U2-1 expression from stage I of lung cancer development contrasts with the recent finding that miR-U2-1 is up-expressed only from stage II in colorectal cancer [31]. Thus, it appears that miR-U2-1 deregulation may be a relatively early event in lung carcinogenesis, reinforcing its potential utility as a lung cancer marker in a screening context. Of particular clinical interest, primarily because a screening test for lung cancer would be of greatest utility when used in a population of patients at risk for lung cancer such as smokers, the miR-U2-1 level appears to be able to discriminate between patients with COPD and patients with COPD and lung cancer with a sensitivity and specificity of 70.6 and 95.5% respectively, and an AUC of 0.866. Thus, in a population of smokers at high risk for lung cancer, miR-U2-1 could be invaluable as a cost-effective way to identify patients who are likely to benefit from more expensive diagnostics, such as spiral CT scanning [63]. While miR-U2-1, by virtue of the promising preliminary data presented here, deserves further study as a lung cancer biomarker, more data are needed before it is ready for validation as a clinical tool. In particular, additional markers need to be identified which, when combined with miR-U2-1 in a multiplexed expression signature, can increase the sensitivity and specificity for lung cancer. Candidate markers have already been identified and multivariable analyses are being applied to determine which candidate markers contribute to the predictive value of the signature with statistical significance. Once such discovery work is complete, the resulting miR-U2-1-based signature must be tested in an independent clinical cohort to confirm its clinical utility and predictive power. Nonetheless, the potential of miR-U2-1 to address an urgent medical need – that of accurately identifying patients with early lung cancer so that they can be targeted for potentially curative surgical resection – is highly significant.

**Table 3:** Overview of demographic and histopathologic data for serum samples included in the cohort.

		Age			Gender		Smoking Status				Histology			Stage Group			
	Number	Mean Age (years)	Median Age (years)	Age Range (years)	Male Number	Female Number	Current Smokers	Former Smokers	Never Smokers	na	Adenocarcinoma	Squamous cell carcinoma	SCLC & Others	Stage I	Stage II	Stage III	Stage IV
**Controls without Symptoms**	45	47	45	30–67	29	16	11		34								
**COPD-Not-Lung Cancer**	22	64	65	45–80	17	5	6	15		1							
**Other Lung Disease-Not-Lung Cancer**	29	44	40	18–80	12	17	4	2	12	11							
**COPD-And-Lung Cancer**	17	67	67	51–85	12	5	12	5			9	7	1	6	3	4	3
**Lung Cancer-Not-COPD**	45	66	64	32–77	33	12	12	22	5	6	23	9	13	8	7	13	16

Detailed information including Ct values is given in Table S3.

## Supporting Information

Figure S1 **Sequence comparison of human U2 snRNA Genes.**
(TIFF)

Figure S2 **Normalized number of reads mapped along the RNU2-2 gene.**
(TIFF)

Figure S3 **miR-1290 is potentially encoded at two locations along the human genome.**
(TIFF)

Figure S4 **Unsupervised hierarchical clustering of the expression of 196 microRNAs.**
(TIFF)

Figure S5 **Anova of miR-U2-1 in the serum of the cohort (Normalized to Cel-miR-39).**
(TIFF)

Figure S6 **Roc (Receiving Operator Curves) of miR-U2-1 in the serum of the cohort (Normalized to Cel-miR-39).** “0” stands for control individuals, “1” for lung cancer patients.
(TIFF)

Figure S7 **The amount of miR-U2-1 in serum varies according to the development stage of lung cancer.**
(TIFF)

Table S1 **Comparison of the number of RPM detected for miR-U2-1 and several canonical microRNAs.**
(XLSX)

Table S2 **Evidence that miR-1290 is a cross-mapping error.**
(XLSX)

Table S3 **Detailed information about demographic, histopathologic data and Ct values for serum samples.**
(XLSX)

Table S4 **Microarrays expression profiles of the 196 microRNAs showing a significant signal.**
(XLSX)

Table S5 **qRT-PCR quantify several isoforms simultaneously.**
(XLSX)

Table S6 **qRT-PCR is less sensitive to additional nucleotides at the 5′ and 3′ ends, than to internal mutations.**
(XLSX)

Table S7 **Sequence reads matching RNU2-1.**
(XLSX)
