# Supplementary figures and images for: Alternative Processing of the U2 Small Nuclear RNA Produces a 19–22nt Fragment with Relevance for the Detection of Non-Small Cell Lung Cancer in Human Serum
Source: PLoS One. 2013 Mar 20;8(3):e60134. doi: 10.1371/journal.pone.0060134 (PMC3603938; doi:10.1371/journal.pone.0060134)

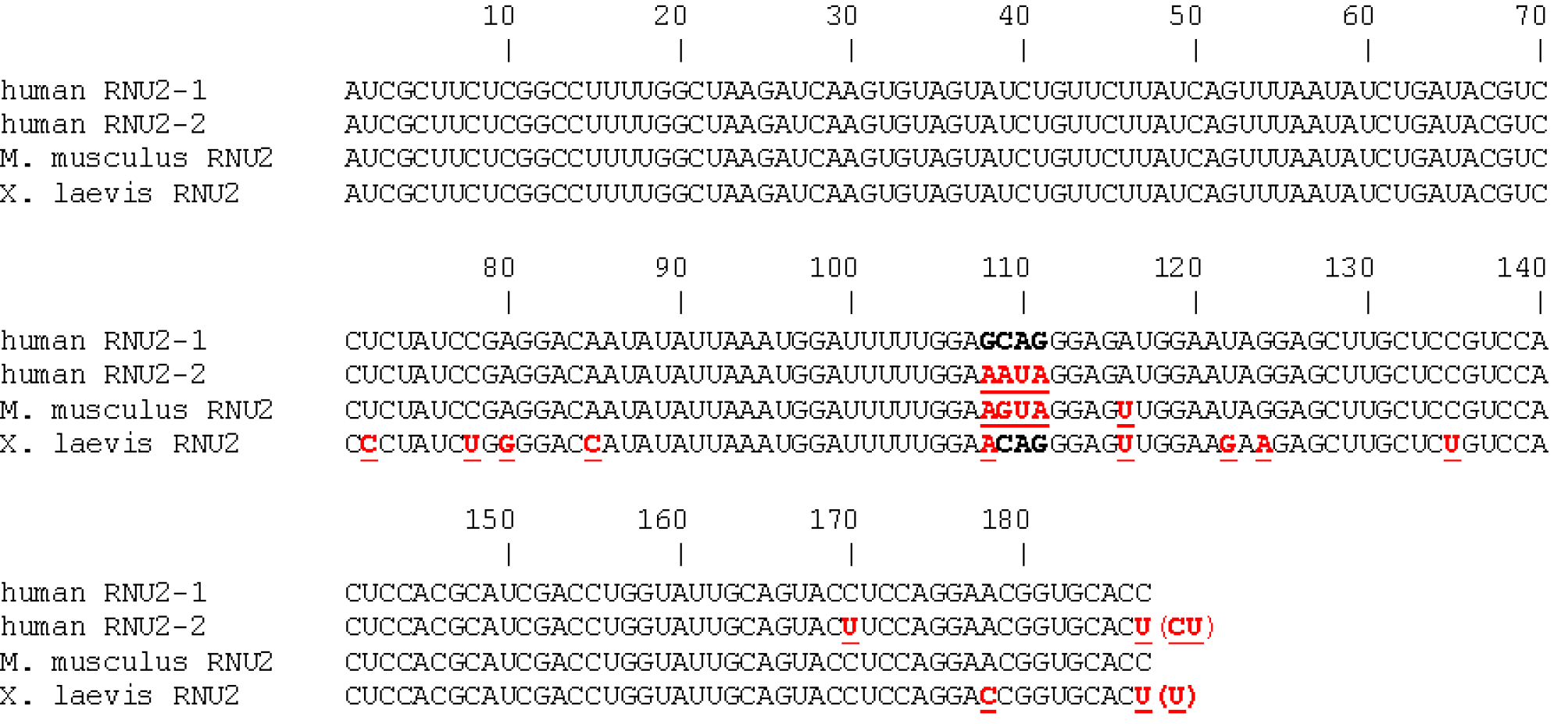

Supplement: Figure S1 — Sequence comparison of human U2 snRNA Genes. (TIFF) [file pone.0060134.s001.tiff]

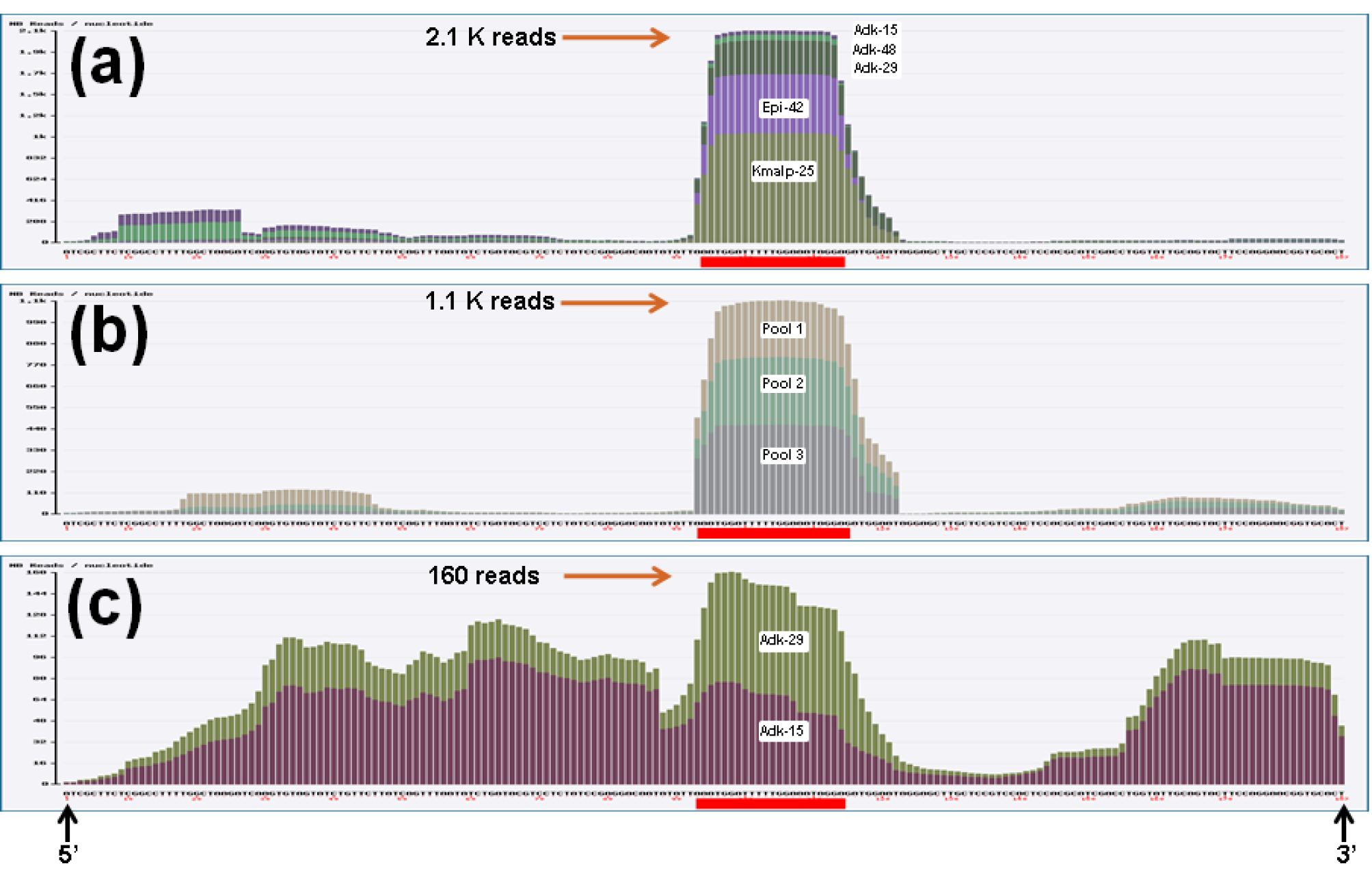

Supplement: Figure S2 — Normalized number of reads mapped along the RNU2-2 gene. (TIFF) [file pone.0060134.s002.tiff]

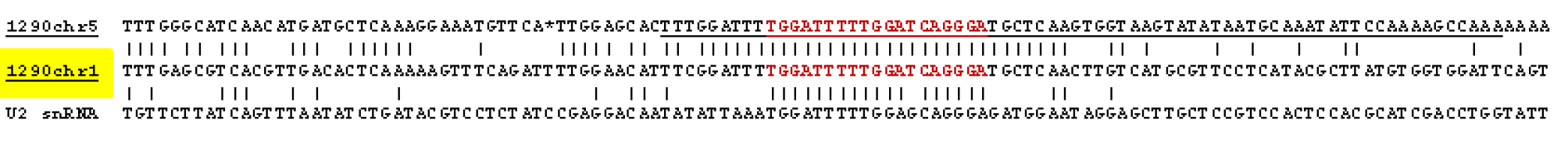

Supplement: Figure S3 — miR-1290 is potentially encoded at two locations along the human genome. (TIFF) [file pone.0060134.s003.tiff]

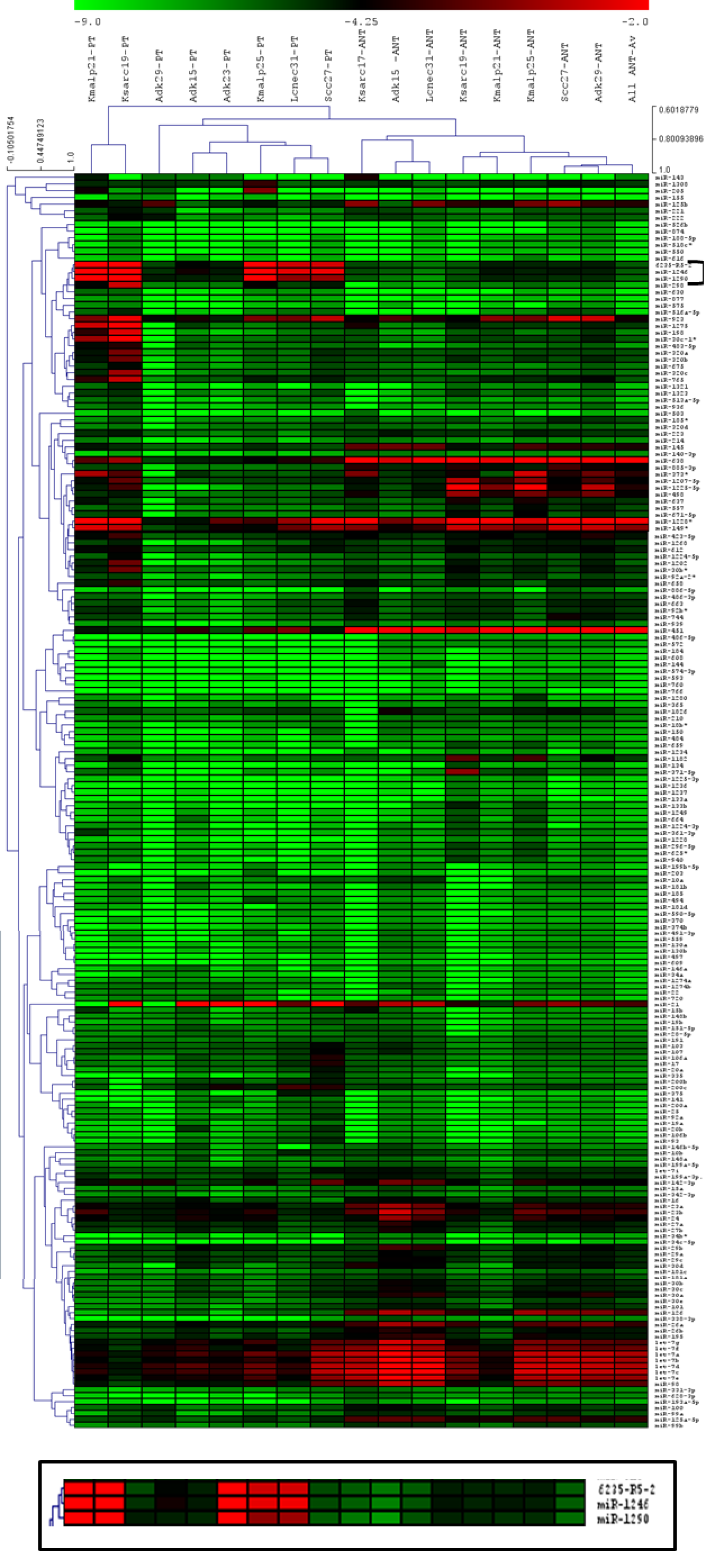

Supplement: Figure S4 — Unsupervised hierarchical clustering of the expression of 196 microRNAs. (TIFF) [file pone.0060134.s004.tiff]

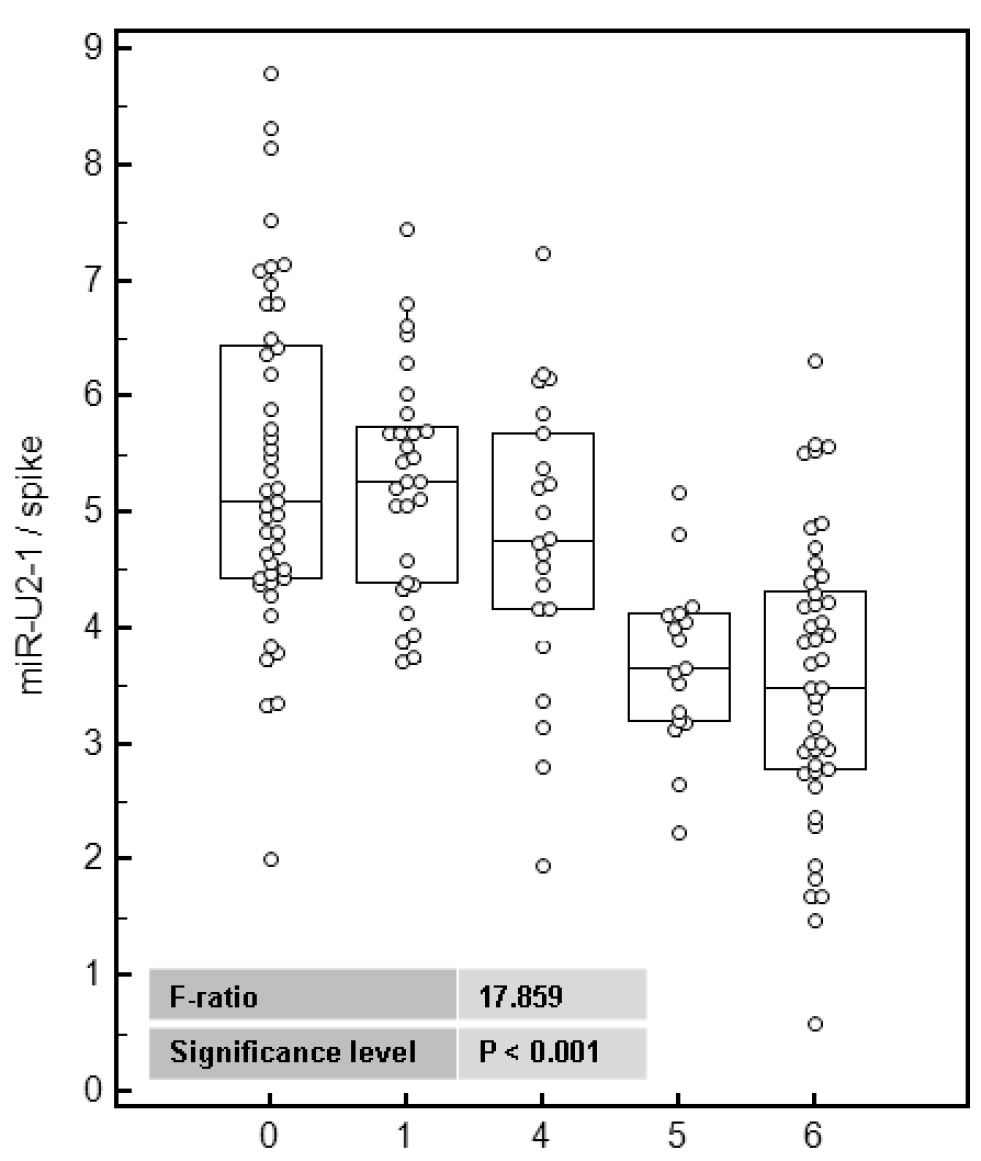

Supplement: Figure S5 — Anova of miR-U2-1 in the serum of the cohort (Normalized to Cel-miR-39). (TIFF) [file pone.0060134.s005.tiff]

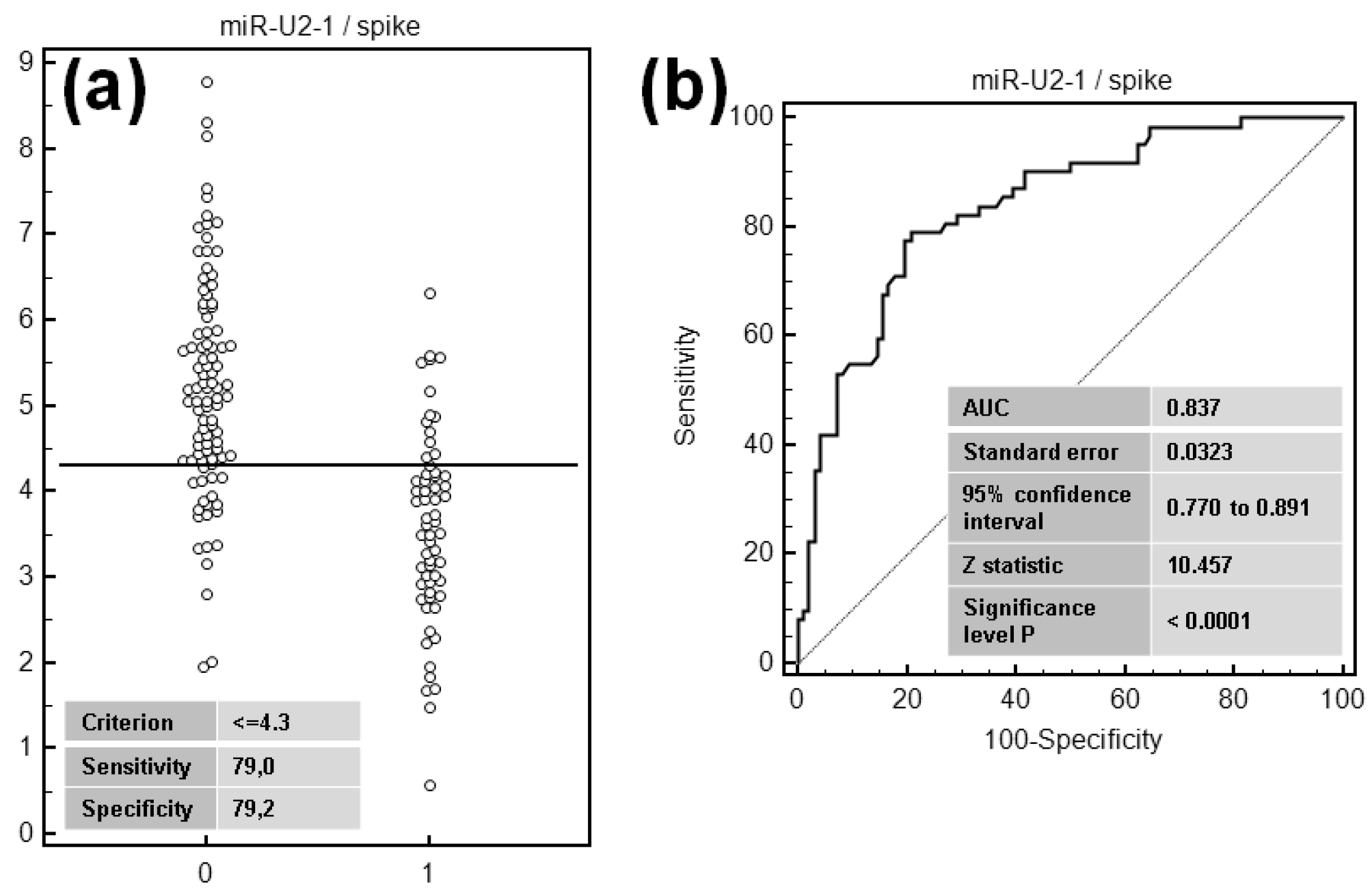

Supplement: Figure S6 — Roc (Receiving Operator Curves) of miR-U2-1 in the serum of the cohort (Normalized to Cel-miR-39). “0” stands for control individuals, “1” for lung cancer patients. (TIFF) [file pone.0060134.s006.tiff]

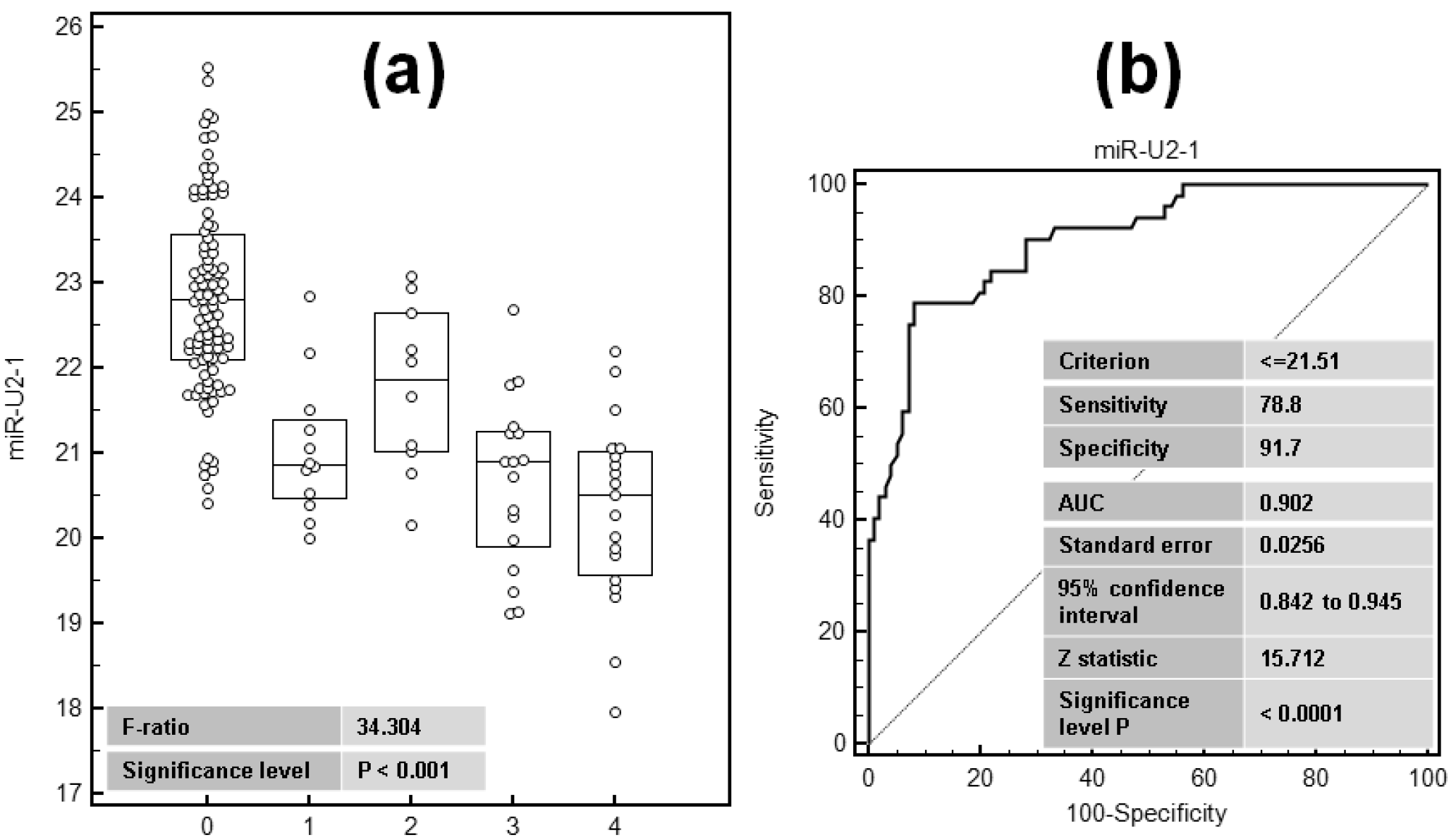

Supplement: Figure S7 — The amount of miR-U2-1 in serum varies according to the development stage of lung cancer. (TIFF) [file pone.0060134.s007.tiff]
